# Cell-Based Biohybrid Sensor Device for Chemical Source Direction Estimation

**DOI:** 10.34133/2021/8907148

**Published:** 2021-01-23

**Authors:** H. Oda, K. Kihara, Y. Morimoto, S. Takeuchi

**Affiliations:** Department of Mechano-Informatics, Graduate School of Information Science and Technology, The University of Tokyo, Japan

## Abstract

This paper describes a method to estimate the direction from which the signal molecule reaches the sensor by using living cells. In this context, biohybrid sensors that utilize a sophisticated sensing system of cells can potentially offer high levels of chemical-detection sensitivity and selectivity. However, biohybrid-sensor-based chemical-source-direction estimation has not received research attention because the cellular response to chemicals has not been examined in the context of directional information. In our approach, we fabricated a device that can limit the interface between the cell-laden hydrogel and the chemical solution of interest to enhance the time difference over which the chemical solution reaches the cells. Chemical detection by cells that express specific receptors is reflected as the fluorescence of the calcium indicator within the cells. Our device has eight chambers that each house 3D cell-laden collagen hydrogels facing circularly outward. The device also works as a cover to prevent chemicals from permeating the hydrogel from above. In our study, by observing the time course of the fluorescence emission of each chamber, we were able to successfully estimate the chemical-source direction within an error range of 7–13°. Our results suggest that a combination of microstructure devices embedded with living cells can be used to exploit cell functionalities to yield chemical-source directional information.

## 1. Introduction

The detection and identification of chemicals in the environment are essential in many situations, particularly in the context of medical tests and field monitoring [1–3]; in medicine, many chemicals are investigated as markers for diagnostics, and as regards field testing, industrial sites are monitored regularly for chemical leaks. These applications require both the ability to distinguish between different chemicals and the estimation of the direction of origin of the chemical source. Detection systems equipped with these capabilities can be used for various applications such as detecting leakage sites and locating various types of creatures in a given environment.

The currently used chemical sensors such as surface plasmon resonance (SPR) [4, 5] and quartz crystal microbalance (QCM) sensors [6, 7] offer highly sensitive detection capabilities. In these sensors, in addition to the sensitivity, the sensor selectivity to chemicals relies on a sensitive membrane positioned on the sensing unit. The membrane is designed to react with specific chemicals, and consequently, changes in the membrane's physical characteristics are exploited for detection. Although there have been significant advances in the fabrication of the sensitive membranes, it is still a challenge to detect chemicals with similar chemical properties with these membranes.

On the other hand, biological cells have a sophisticated sensing mechanism that allows for the highly selective detection of chemicals through their membrane proteins [8, 9]. Some membrane proteins can even distinguish optical isomers and chemicals with different carbon molecules. Membrane receptors themselves have been used to increase the selectivity of sensitive membranes in conventional sensors [5, 7, 10, 11]. Moreover, with emerging technologies in biology, the receptor of choice can be genetically transfected into cells. As regards the detection mechanism, we note that the binding of analytes to receptors increases the calcium-ion concentrations in the cytoplasm [12, 13]. Via the detection of the change in the calcium-ion concentration, cells with receptor proteins of choice can be used as sensor elements with both high selectivity and sensitivity; such cell-based biohybrid sensors have previously been the topic of our research [14–16]. However, cell-based biohybrid sensors for chemical-source-direction estimation have not been considered for practical application because cellular responses to chemicals do not contain directional information. Therefore, a biohybrid device with multiple cell-based sensor units that only respond to a specific direction is needed for direction estimation.

**Figure 1:**
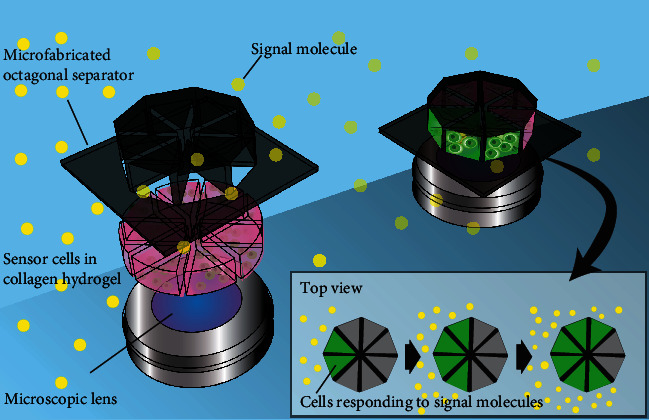
Schematic of the proposed cell-based biohybrid sensor. Collagen hydrogel embedded with sensor cells is placed in each chamber of the microfabricated octagonal separator device. The fluorescence emission of the calcium indicator in the cells reacting to the chemical of interest is detected through a microscopic lens. The direction of the chemical source can be calculated by observing the time course of the fluorescence change in each chamber.

In this study, we develop an octagonally shaped sensor with eight separate chambers containing cell-laden hydrogel to estimate the chemical-source direction, as illustrated in Figure 1. The device has a limited number of openings to the outside environment, which aids in limiting the arrival of the analytes to the cells in each chamber. By measuring the arrival-time difference between each chamber of the octagonal sensor device, it is possible to estimate the direction of the chemical source. As a demonstration, we prepare a cell-based biohybrid sensor device composed of multiple octagonal sensors embedding cells with muscarinic acetylcholine receptors and visualize the cellular response to muscarine molecules using a calcium indicator. The muscarine-source direction is estimated by analyzing the response time of the cells in each chamber of the device.

## 2. Results

### 2.1. Fabrication of Cell-Based Biohybrid Sensor Device to Detect Chemical-Source Direction

**Figure 2:**
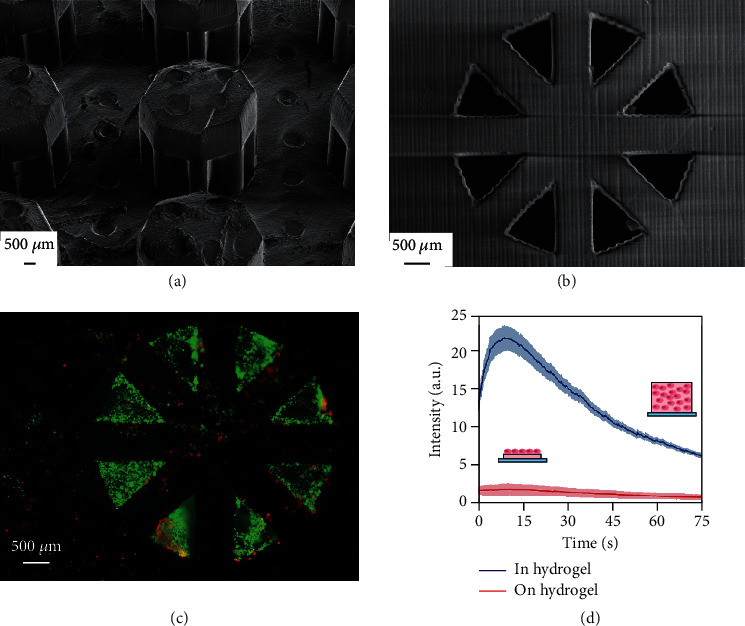
(a) Scanning electron microscope (SEM) image of octagonal separators on the sensor platform. With the supporting parts for the 3D-printing process positioned at the top surface of the device, we were able to prepare a platform with a smooth bottom surface that contacted the glass bottom dish. (b) SEM image of the octagonal separator from the bottom side. Eight openings were created in an octagonal formation. (c) Fluorescence image of LIVE/DEAD staining cells in the octagonal sensor. (d) Temporal variation in the fluorescence intensity emitted from cells in reaction to muscarine depending on culture conditions: monolayered culture (red) and 3D culture (blue). The intensity was higher for the 3D-cultured cells inside the hydrogel than for the monolayered cells on the hydrogel. The intensity change was also clearly visible for the cells in the hydrogel when compared with the cells on the hydrogel.

We fabricated a sensor platform composed of 9 octagonal separators using a 3D printer. From the scanning electron microscope (SEM) images of the sensor platform (Figures 2(a) and 2(b)), we confirmed that all the octagonal separators were fabricated as designed. The openings were limited to the bottom and the lateral side facing outward. In line with our design, the smooth surface of the sensor platform afforded tight sealing between the glass-bottomed dish and the octagonal separator and enabled chemical contact with cells only from the openings on the separator's outer side.

After embedding collagen with human embryonic kidney cells with stable expression of muscarinic acetylcholine receptors (HEK293T M5C6) cells in the octagonal separators, we stained the cells with calcein AM and ethidium homodimer to assess the cytotoxicity of the 3D-printeddevice (Figure 2(c)). Most of the cells exhibited green fluorescence, indicating that they were live cells. Of the total fluorescence area, 82.0% corresponded to green fluorescence; this value did not significantly differ from the value of the green fluorescence area calculated from the 3D-cultured cells inside collagen hydrogel without the device (82.2%). These results suggest that the cells inside the collagen hydrogel are not damaged either from the introduction of cells into the chamber or from any toxins diffusing out from the 3D-printed device.

To compare the fluorescence intensities emitted from monolayered and 3D-cultured HEK293T M5C6 cells, we prepared cells cultured on collagen hydrogel and cells embedded in collagen hydrogel. In both cases, changes in the intensity were confirmed during muscarine application to the cells. As shown in Figure 2(d), the fluorescence intensity increases after muscarine application to the cells, reaching a peak at ~10 s after the application of muscarine. We speculate that the subsequent decrease in the fluorescence intensity after the peak is due to the cellular regulation system that maintains the calcium-ion concentration at a constant level. Even though the cells corresponding to the two abovementioned conditions exhibited similar intensity-profile trends, the intensity of the embedded cells was significantly higher than that observed from the cells on the collagen hydrogel. In this regard, previous reports have indicated that the fluorescence intensity observed with a fluorescence microscope increases when cells are embedded in a 3D structure because of the vertical accumulation of cellular fluorescence [14]. The results obtained in this experiment also indicate that our proposed device exhibits greater sensitivity to chemical detection than single cells or monolayer cells.

### 2.2. Chemical Detection

Prior to the application of a target chemical solution to the cell-based biohybrid sensor, we confirmed the morphology of the flow patterns of the chemical solution. By visualizing the flows using ink, we confirmed that the flow spread evenly around the release port of the ink-flow device (Supplemental Figure 1(a)). With these data, we defined the direction of the release port as the direction of the chemical sources for further experiments.

**Figure 3:**
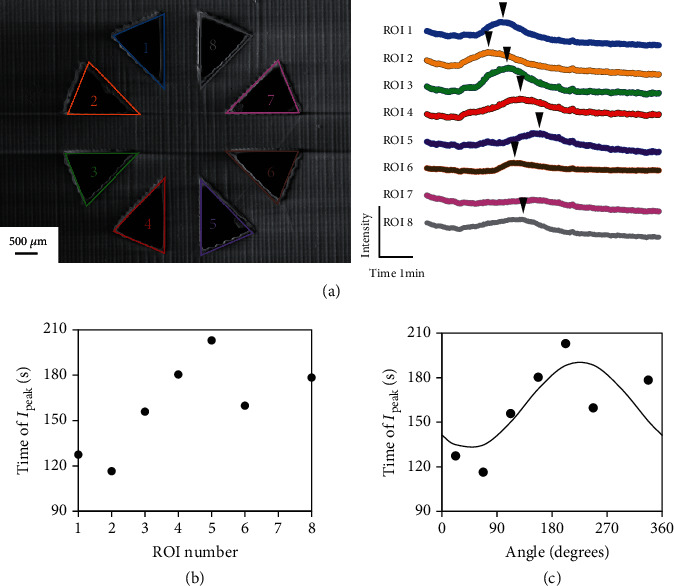
(a) Changes in fluorescence intensity at each region of interest (ROI) of the cell-based biohybrid sensor. The ROI numbers were assigned as shown in the image. The black arrow indicates the timing of peak detection. (b) Time of peak intensity (*I*_peak_) at each ROI. (c) Curve fitted by means of a sinusoidal approximation to the time of peak intensity. Each ROI number was converted into the corresponding angle, and subsequently, the angle that minimized *I*_peak_ according to the curve was estimated as the direction of the chemical source. Scale bar: 500 *μ*m.

Next, we applied the muscarine solution to the cell-based biohybrid sensor. The fluorescence intensity during muscarine application was observed at each region of interest (ROI) delineated in each chamber (Figure 3(a)). For each ROI, we calculated the time corresponding to the peak fluorescence intensity, which was defined as the first intensity value that was 1.65 times the average intensity before and after 70 frames of observation. Although the peak intensity was different for each ROI owing to the difference in cell numbers and the range of cellular functions, the time of peak intensity can be determined as a single point in each case; the time of peak intensity was considered as the time corresponding to *I*_peak_ (Figure 3(b)). The calculated *I*_peak_ times and the curve fitted by means of the sinusoidal approximation were plotted against the angle defined by the corresponding ROI number, as shown in Figure 3(c). The direction with the lowest fitting value of the time was then estimated as the chemical-source direction. Consequently, our cell-based biohybrid sensor estimated the chemical-source direction to be 34°, whereas the actual direction of the muscarine source was 45°.

To examine the effectiveness of the device, we compared the analyte detection of the cell-laden collagen hydrogel with and without the octagonal separator. Supplemental Figure 2 shows the changes in the intensity of a cell-laden octagonal hydrogel without the separator. The time course of the intensity does not show a significant difference from ROI to ROI. Moreover, the defined peak intensity is only detected in one ROI. Because there is no cover on the hydrogel, which limits the analyte diffusion into the hydrogel from the topside, the analyte is detected by the cells over various time intervals, resulting in the broad intensity profile.

**Figure 4:**
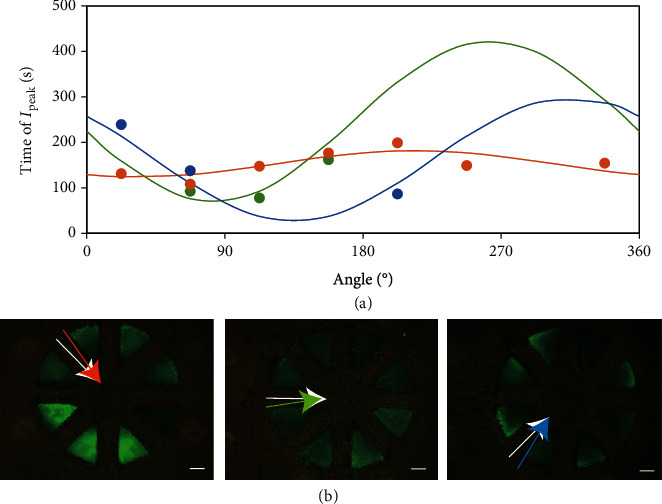
(a) Relationship between the assigned angle corresponding to each region of interest (ROI) and the time to peak intensity (*I*_peak_) at each ROI. The curves were fitted by sinusoidal approximation to the plots. The actual experimental data are indicated by the dots, and the fitting is indicated as the curves. The colors orange, green, and blue represent the data relating to chemical-source-direction angles of 45°, 90°, and 135°, respectively. (b) Fluorescence images of the cell-based biohybrid sensor 210 s after applying muscarine using the device for chemical-solution flow. White arrows indicate the direction of the applied chemical, whereas the colored arrows indicate the estimated direction. These colors correspond to the counterpart ones used in (a). Scale bar: 500 *μ*m.

To demonstrate the efficacy of our cell-based biohybrid sensor in determining the chemical-source direction, we positioned a chemical source along three different directions: 45°, 90°, and 135°.  Figure 4(a) shows the calculated time of “chemical arrival” and the fitting curve for each case to estimate the chemical-source direction. We note that the cell-based biohybrid sensor reasonably closely estimates the chemical-source direction (estimated direction: 34°, 97°, and 146°; actual direction: 45°, 90°, and 135°, respectively) (Figure 4(b)). In the study, the number of ROIs used for estimation was different for each experimental scenario. Regardless of the number of ROIs, the estimated chemical-source direction exhibited an average error in the range of 7–13°.

## 3. Discussion

We developed a cell-based biohybrid sensor device to estimate the chemical-source direction by observing the reaction of HEK293T cells expressing muscarinic acetylcholine receptors to muscarine. In our device, by limiting the number of openings to each chamber to the outward-facing side and limiting the chemical-solution-interface of the cell-laden hydrogel, we were able to focus on the difference in arrival time of the chemical solution. Moreover, the 3D arrangement of cells in the ROIs enhanced the detected fluorescence, and the averaging of the signals from each cell ensured noise reduction. This situation enabled data analysis with a sinusoidal curve fitting for chemical-source-direction estimation with a standard deviation of 11.9°.

When compared with industrial-grade sensors, the cell functionalities in our device exhibit massive variations. In chemical detection through membrane receptors, the number of receptors on the cells can vary in number, which can form a drawback of cell-based biohybrid sensors. However, our approach integrates the signals obtained from a large number of cells to a uniform signal. Via the averaging of the signals from many cells, the variation pertaining to each cell could be eliminated, and a single large-amplitude signal could be used for estimation; this aspect is significant because there can be a maximum of ~10^6^ cells in each chamber. Nevertheless, we note here that very high cell concentrations can suppress the diffusion of chemicals inside the hydrogel. In this study, we limited the number of cells in each section to 10^5^ cells. The fluorescence of the cells was observed for the entire section, which indicated the occurrence of a sufficient amount of chemical diffusion.

We also estimated the flow speed based on the ink flow, as shown in Supplemental Figure 1(b). We note that it requires ~20 s for the chemical-solution flow to traverse one octagonal device. On the other hand, the difference in the *I*_peak_ time is ~100 s (Figures 2 and 3). We hypothesize that three factors underlie the observed time difference between the chemical arrival at the interface and the fluorescence observation: chemical diffusion through the collagen hydrogel matrix, calcium-ion-concentration increase following the reaction to a receptor, and the increase in the reacted calcium indicator to the detectable level. The chemical flow and reaction number of calcium indicators appear to be the primary factors that increase the reaction time as the biological reaction itself occurs in less than 100 ms [17], which can therefore be neglected.

The estimated-direction error was smaller than the opening angle of each chamber (45°). For the chemical-source direction estimated from the ROI position that exhibits the “earliest” observed peak intensity, the resolution of the detection angle is limited to the opening angle of the chamber. The use of the sinusoidal approximation to estimate the chemical-source direction allows for more precise detection with fewer ROIs. In general, increasing the number of ROIs will increase the estimation accuracy. In our case, increasing the number of sides of the polygonal sensor affords a decrease in both the number of cells in each section and the interior angles. We believe that there is a limit to which the number of sides of the polygonal sensors can be effectively increased.

There are two basic approaches to chemical-source-direction estimation. One is to use a movable sensor, wherein its mobility facilitates the sensor to compare the signal-intensity difference to estimate the chemical-source direction [3, 18]. The estimation accuracy at a single position is not very high for such devices; however, the device accuracy can be improved by acquiring measurements over a number of points. A second approach is to use an array of sensors and estimate the direction of the chemical source from the difference in the reaction times [19–21]. Such an approach requires an array of sensors and detectors to obtain data, which results in complicated detection systems. Many studies have used a similar strategy to estimate the source direction of sounds and sonar signals [22, 23]; however, few studies have focused on such strategies for arrayed cell-based biohybrid sensor systems. Our approach involving the use of microstructures to restrain the chemical-solution-flow direction to the array of cells necessitates the use of only one fluorescence imaging system to estimate the chemical-source direction based on the difference in the analyte arrival time to each ROI. Our results indicate that the combination of microstructure devices with cells affords multiple benefits based on the unique functionality of cells. Although we only observed the fluorescence through microscopy, we note here that the size of each cell-based biohybrid sensor is sufficiently small to enable its mounting on a portable sensing system, and the fabrication of chambers with 3D-printed walls ensures identically shaped ROIs for all the samples. This reproducibility makes it easier to automate the analysis process.

## 4. Materials and Methods

### 4.1. Cell Culture

Human embryonic kidney cells with the stable expression of muscarinic acetylcholine receptors (M5 with C-terminal HA Tag Stable Expressing HEK 293T Cell Line-Clone C6(HEK293T M5C6)) were used as model sensor cells in this study [24–26]. HEK293T M5C6 cells were obtained by using a lentivirus system (Applied Biological Materials, Canada). The cells were stored in a Cellbanker (Takara Bio Inc., Shiga, Japan) with a concentration of 1.0 × 10^6^ cells/mL. Once the cells were thawed, they were cultured in Dulbecco's modified Eagle's medium (DMEM) (Sigma-Aldrich, USA) containing 10% (*v*/*v*) fetal bovine serum (FBS) (Biosera, France), 100 U/mL penicillin, 100 *μ*g/mL streptomycin (Sigma-Aldrich, USA), and 0.8 *μ*g/mL of puromycin (Sigma-Aldrich, USA). The cells were split before confluence and used in the experiment after no more than 30 passages.

### 4.2. Design and Preparation of Device for Chemical-Solution Flow

As an experimental setup to demonstrate chemical-source-direction estimation using the proposed device, we fabricated a device to release a chemical solution (the chemical-solution-flow device). The device was mounted on a dish and able to eject a chemical solution from a release port 8.5 mm away from the center of the dish, thereby leading to the generation of chemical-solution flow from the port in the dish (Supplemental Figure 1). The chemical-solution flow was also observable owing to a window opened near the center of the device to allow access to a microscope. The device was prepared with a 3D printer (AGILISTA-3000, Keyence, Japan) and subsequently washed with water to remove the resin on its surface.

### 4.3. Design and Preparation of Cell-Based Biohybrid Sensor Device for Estimation of Chemical-Source Direction

**Figure 5:**
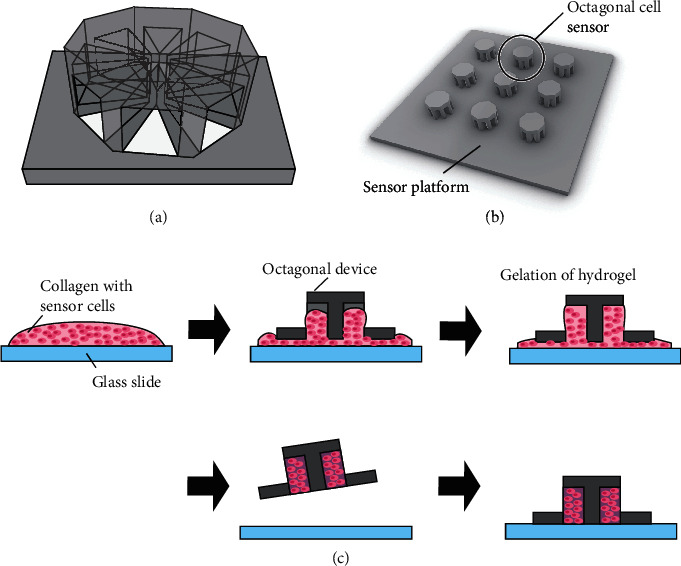
(a) Schematic illustration of the octagonal separator. (b) Computer-aided design of the sensor platform containing octagonal separators. Multiple octagonal separators were placed on the center of the square platform. (c) Procedure for cell introduction into the octagonal separator. Collagen with sensor cells was placed on the glass slide, and subsequently, the sensor platform was placed on top to fill each chamber with cell-laden collagen. After incubation to confirm the gelation of collagen, the excess collagen was removed from the device.

In our study, we designed an octagonal separator for our sensor, as shown in Figure 5(a). The octagonal separator was designed to have a diagonal length of 2.5 mm, which fitted the field of view of a microscope (IX71, Olympus, Japan) equipped with a 4x objective lens and loaded with the associated software (CellSens, Olympus). The octagonal separator had eight triangular chambers, with openings to the bottom and outer sides. The opening at the bottom was used for embedding the hydrogel with cells, and the opening on the outer side was the only opening facing the environment because we set this device on a glass-bottom dish. The height of each chamber was 1.5 mm.

Multiple octagonal separators were placed on one sensor platform to have octagonal device in different direction from the release port (Figure 5(b)). The platform was square-shaped, with a side length of 18 mm. The octagonal separators were placed at the center of the platform in a smaller square format. The platform was designed to fit the bottom of the 35 mm-per-side glass-bottom dish to ensure that it could be placed in the same position for each trial. Three octagonal sensor devices on the side nearest to the release port of the chemical-solution-flow device were chosen as measurement sensors for each experiment. The three sensors were angled at 45°, 90°, and 125° from the release port of the chemical-solution-flow device. The sensor platform was fabricated by using a stereolithography machine (J028, DWS) with a black resin (DL380, DigitalWax®, DWS). After we washed the platform with ethanol to remove the remaining resin on its surface, it was coated with 2 *μ*m thick parylene C and sterilized by UV irradiation.

The process of embedding cells in the chambers is illustrated in Figure 5(c). Collagen was first mixed with 10x Hanks' balanced salt solution (HBSS) buffer, and its pH was adjusted to 7.4 with the use of NaHCO_3_ solution on ice. HEK293T M5C6 cells were collected and resuspended in collagen solution. Next, 100 *μ*L of cell-suspended collagen solution was quickly placed on a cell culture dish, and the platform with the octagonal separators was pushed against the collagen solution to fill the chambers. The excess amount of collagen at the bottom was scraped off, and the platform was placed in a glass-bottomed dish (side length of 35 mm). The entire setup was placed in an incubator operated at 37°C to ensure the complete gelation of the collagen. Finally, we obtained a cell-based biohybrid sensor. Culture medium (2 mL) was added to the culture dish and incubated overnight before the chemical-detection experiments were performed. Across all experimental trials, 25.7% of the trials had all eight chambers filled with cell-laden hydrogels. As regards the devices, 68.6% of the devices had more than three chambers with cells, which were used for analysis.

To compare the reaction of cells with and without the chambers, we also prepared a cell-laden octagonal hydrogel sample. The preparation step for this sample was identical to that for the octagonal device. We prepared a chamber-less octagonal cast device of the same dimensions as the proposed device. The collagen hydrogel with cells was prepared in this device on a collagen-coated glass-bottom plate. After we confirmed the gelation of the hydrogel, the casting device was removed from the dish to leave intact the octagonal hydrogel without any device.

Next, we applied a calcium indicator (Fluo-8AM, AAT Bioquest, USA) to the cells in the cell-based biohybrid sensor to visualize changes in the calcium concentration related to chemical recognition through its receptor [18, 19]. Fluo-8AM was dissolved in dimethyl sulfoxide (DMSO) diluted with 20 mM-HEPES-buffered HBSS (pH = 7.2) to a final concentration of 5 *μ*M. The Fluo-8 solution was added to the 35 mm-per-side dish with the cell-based biohybrid sensor and incubated for 30 min. It was subsequently washed with HEPES-buffered HBSS twice and removed with 2 mL of HBSS solution before chemical application.

When checking the viability of the cells in the device, we applied a solution containing Hoechst 33342, 2 *μ*M of calcein AM (Thermo Fisher, USA), and 3 *μ*M ethidium homodimer (Thermo Fisher, USA). After the device was incubated for 15 min, it was washed with HEPES-buffered HBSS twice and observed through a fluorescence microscope to check for the cell viability inside the cell-based biohybrid sensor. For comparison, we performed the same test on the cells in the collagen hydrogel without the device. Fluorescence images from both sets of cells (with and without the device) were analyzed by using the ImageJ software (NIH, Bethesda, MD, USA) to calculate the area of fluorescence.

### 4.4. Application of Chemical Solutions

Via silicone tubes, we connected a syringe pump (KDS-210, KD Scientific Inc., USA) to the device for chemical-solution flow. For the preparation of a target chemical solution, muscarine chloride (Sigma-Aldrich) was dissolved in HEPES-buffered HBSS solution at a concentration of 10 *μ*M. The detection range of muscarine chloride was 1.0 *μ*M to 300 *μ*M (Supplemental Figure 3). The muscarine solution was released from the device for chemical-solution flow at a flow rate of 0.5 mL/min and applied to the cell-based biohybrid sensors. At this flow rate, the Reynolds number was calculated as 3.3, and the chemical-solution flow reached the back surface of the octagonal device. We observed the cell-based biohybrid sensors under the muscarine solution flow through a microscope equipped with a 4x objective lens and loaded with the associated software, and we recorded the green fluorescence for 7 min with a frame rate of 2.7 fps. In this experiment, we only used the device for a single measurement because the fluorescence of calcium indicator fades (Supplemental Figure 4) during continuous exposure.

### 4.5. Chemical-Source-Direction Calculation

The ROI was set to each triangular chamber of the octagonal sensor device by using the ImageJ software (NIH, Bethesda, MD, USA). Using ImageJ, we calculated the fluorescence intensity of each ROI for each frame. Before chemical detection, we measured the fluorescence change of the octagonal sensor with no chemicals to measure the fluorescence curve for photobleaching (Supplemental Figure 3). The fluorescence intensity (*I*) of each ROI at time *t* was fitted to the following equation:

(1) $$\begin{array}{c} b\times e^{-at}+c, \end{array}$$

where *a* and *b* were calculated by using Python (SciPy library, curve_fit module). The time course of the fluorescence intensity corresponding to each ROI was modified by subtracting the photobleaching factor by using the abovementioned fitting curve.

As muscarinic acetylcholine receptor reacts to muscarine, calcium ions flow into the cells, inducing an increase in the calcium-ion concentration in the cells. The calcium-ion concentration subsequently declines because of the cell's regulation system, which maintains the calcium-ion concentration at a constant level. Thus, the fluorescence intensity of the sensor cells peaks during signal recognition. We considered the time of this peak intensity as the representative time for chemical detection for each ROI; the time of the peak fluorescence intensity was defined as the first time when the difference between the average fluorescence intensity in the ROI and the intensity before and after 70 frames increased above the noise level of 1.65 according to the measured fluorescence of the cell-based biohybrid sensor without analytes. These values of 70 frames and 1.65 are valid for our fluorescence microscope. For each ROI, we assigned the angle of the line that runs through the center of the ROI as the reference angle. The relationship between the corresponding angle (*θ* [deg]) of each ROI and the time of peak fluorescence intensity (*t* [*s*]) was plotted and fitted as per the following equation:

(2) $$\begin{array}{c} t=k\sin\left(\frac{\pi }{180}\left(\theta +l\right)\right)+m, \end{array}$$

where *k*, *l*, and *m* denote constants. The angle that affords the minimum *t* value was estimated as the chemical-source direction [27]. The dataset for curve fitting was achieved every 45° due to the separator of the octagonal device. This data taking pattern made it easy to perform sinusoidal curve fitting.

## Supplementary Materials

Supplementary Materials Supplemental Figure 1: (a) Experimental setup of the chemical-solution-flow device and the photograph of the device with solution flow visualized by red ink. (b) Plot of the relationship between the flow rate of the chemical solution and flow speed at the center of the dish. Supplemental Figure 2: changes in the fluorescence intensity at each region of interest (ROI) as obtained from an octagonal hydrogel without the separator. The ROI number was assigned as shown in the figure. The black arrow indicates the timing of peak detection. Supplemental Figure 3: the time course of the intensity changes of the calcium indicator inside the cells with muscarinic acetylcholine receptors in reaction to different concentrations of muscarine. The detection of muscarine was determined by confirming the peak intensity detection (indicated in the black arrow). Supplemental Figure 4: intensity changes in each chamber due to the fading of the fluorescent molecules. These data were acquired without any signal chemicals.
