# Supplementary material for: Cell-Based Biohybrid Sensor Device for Chemical Source Direction Estimation
Source: Cyborg Bionic Syst. 2021 Jan 23;2021:8907148. doi: 10.34133/2021/8907148 (PMC9494699; doi:10.34133/2021/8907148)
Supplement: Supplementary Materials — Supplemental Figure 1: (a) Experimental setup of the chemical-solution-flow device and the photograph of the device with solution flow visualized by red ink. (b) Plot of the relationship between the flow rate of the chemical solution and flow speed at the center of the dish. Supplemental Figure 2: changes in the fluorescence intensity at each region of interest (ROI) as obtained from an octagonal hydrogel without the separator. The ROI number was assigned as shown in the figure. The black arrow indicates the timing of peak detection. Supplemental Figure 3: the time course of the intensity changes of the calcium indicator inside the cells with muscarinic acetylcholine receptors in reaction to different concentrations of muscarine. The detection of muscarine was determined by confirming the peak intensity detection (indicated in the black arrow). Supplemental Figure 4: intensity changes in each chamber due to the fading of the fluorescent molecules. These data were acquired without any signal chemicals. [file 8907148.f1.docx]

Supplementary Materials


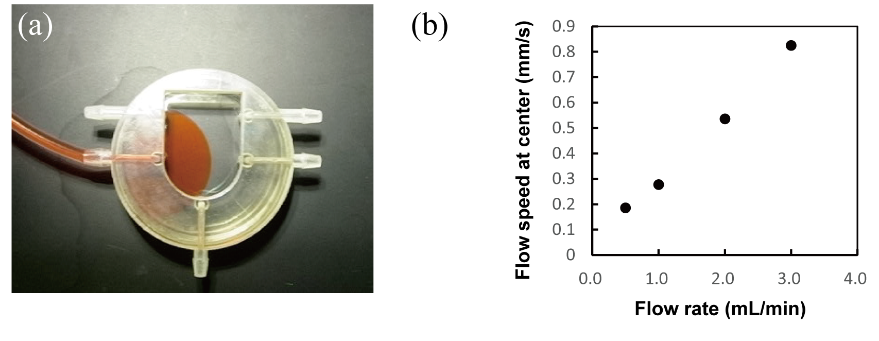


Supplemental Figure 1. (a) Experimental setup of the chemical-solution-flow device and the photograph of the device with solution flow visualized by red ink. (b) Plot of the relationship between the flow rate of the chemical solution and flow speed at the center of the dish.


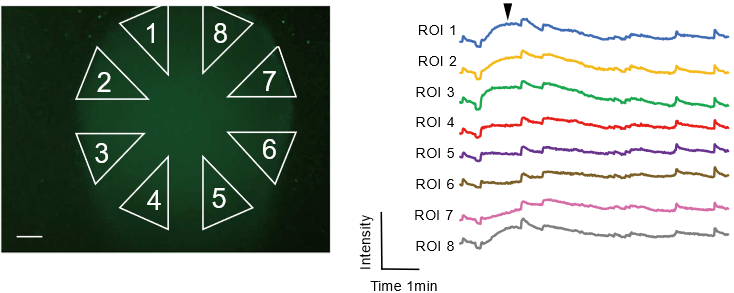


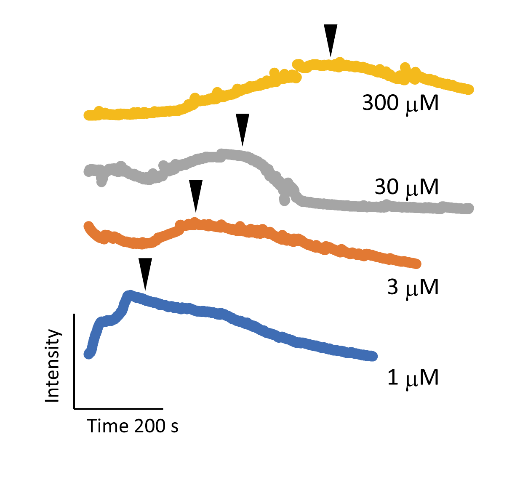
Supplemental Figure 2. Changes in the fluorescence intensity at each region of interest (ROI) as obtained from an octagonal hydrogel without the separator. The ROI number was assigned as shown in the figure. The black arrow indicates the timing of peak detection.

Supplemental Figure 3. The time course of the intensity changes of the calcium indicator inside the cells with muscarinic acetylcholine receptors in reaction to different concentrations of muscarine. The detection of muscarine was determined by confirming the peak intensity detection (indicated in the black arrow).


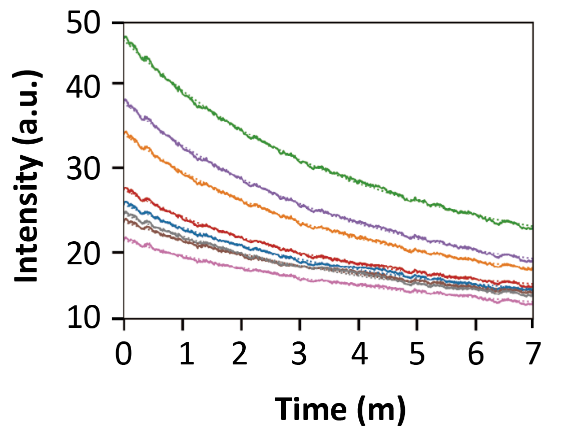


Supplemental Figure 4. Intensity changes in each chamber due to the fading of the fluorescent molecules. These data were acquired without any signal chemicals.
